# Robotic hepatectomy and biliary reconstruction for perihilar cholangiocarcinoma: a pioneer western case series

**DOI:** 10.1007/s13304-021-01041-3

**Published:** 2021-04-16

**Authors:** Umberto Cillo, Francesco Enrico D’Amico, Alessandro Furlanetto, Luca Perin, Enrico Gringeri

**Affiliations:** Hepatobiliary Surgery and Liver Transplantation Unit, Padua University Hospital, 2° Piano Policlinico, Via Giustiniani 2, 35128 Padua, Italy

**Keywords:** Cholangiocarcinoma, Robotic surgery, Major liver resection, Learning curve

## Abstract

Open surgery is the standard of care for perihilar cholangiocarcinoma (pCCA). With the aim of oncologic radicality, it requires a complex major hepatectomy with biliary reconstruction. The postoperative course is consequently often complicated, with severe morbidity and mortality rates of up to 27.5–54% and 18%, respectively. Robotic liver surgery is emerging as a safe, minimally-invasive technique with huge potential for pCCA management. After the first case described by Giulianotti in 2010, here we present the first western series of robot-assisted liver resections with biliary reconstruction for pCCA with the aim to preliminarily assess the feasibility and repeatability of the procedure. At our high-volume teaching hospital center dedicated to HPB surgery, 128 pCCA patients have been surgically treated in the last 15 years whereas more than 800 laparoscopic liver resections have been performed. Since the Da Vinci Xi Robotic platform was introduced in late 2018, 6 major robotic liver resections with biliary reconstruction have been performed, 4 of which were for pCCA. All 4 cases involved a left hepatectomy with caudate lobectomy. The median operating time was 840 min, with a median blood loss of 700 ml. One case was converted to open surgery during the reconstruction due to a short mesentery preventing the hepatico-jejunostomy. None of the patients experienced major complications, while minor complications occurred in 3 out of 4 cases. One biliary leak was managed conservatively. The median postoperative stay was 9 days. Negative biliary margins were achieved in 3 of the 4 cases. An included video clip shows the most relevant technical details. This preliminary series demonstrates that robot-assisted liver resection for pCCA is feasible. We speculate that the da Vinci platform has a relevant potential in pCCA surgery with particular reference to the multi-duct biliary reconstruction. Further studies are needed to better clarify the role of this high-cost technology in the minimally-invasive treatment of pCCA.

**Supplementary Information:**

The online version contains supplementary material available at 10.1007/s13304-021-01041-3.

## Introduction

There is widespread agreement that perihilar cholangiocarcinoma (pCCA) is a biliary duct cancer characterized by an extremely complex surgical management and an unfavorable prognosis if not treated surgically [1, 2]. Radical resection with negative margins is the only curative treatment for this insidious disease. The complexity of pCCA treatment stems from the tumor’s proximity to the hepatic artery and portal vein bifurcations. Its tendency to frequently encase these vessels and its potential for intraductal growth pose further challenges for the surgeon, in terms of either preoperative decision-making or intraoperative management, particularly as regards the need for multi-duct hepatico-jejunostomies.

Postoperative morbidity and mortality rates may be as high as 27.5–54% and 18%, respectively [3].

Innovative minimally-invasive technologies and the increased experience in hepatic laparoscopic surgery have recently made parenchymal transection safer and easier, promoting a more extensive use of minimally-invasive approaches. On the one hand, laparoscopic liver resections have been shown to cause less postoperative pain, and to enable a faster return to oral food intake, and a faster functional recovery. They are associated with a better morbidity profile than open surgery, without jeopardizing long-term oncological results in the treatment of a number of different cancers [4–6]. The proportion of minimally-invasive interventions conducted at high-volume hepatobiliary (HPB) centers has consequently increased dramatically in the last 5 years [7]. On the other hand, laparoscopic approaches have yet to overcome those limitations relating to a lack of dexterity that have proved crucial when performing complex dissections and reconstructions of small structures. As far as pCCA is concerned, the rarity of the disease, the technical complexity associated with the critical location, the frequent anatomical variations, the need for oncologically free margins, and, more importantly, the dexterity needed to perform multi-duct deep-seated hepatico-jejunostomies, have limited the adoption of a laparoscopic approach to its treatment.

Robotic surgery is emerging as a safe technique that combines the advantages of open surgery (3-dimensional view, 7 degrees of freedom) with those of a minimally-invasive approach. It has huge potential for use in the management of pCCA [8]. That said, although robot-assisted liver resections are used more and more at high-volume HPB centers [9], after the first single case description by Giulianotti in 2010 [10] there are still only very few case series published of fully robotic liver resections with hepatico-jejunostomies for pCCA [11–13]. As far as we are concerned, most series are from China and none from the west. Only 2 case reports involving this procedure have been described so far in the literature from the western world [14].

On these grounds, we ran a study to assess feasibility and repeatability of a fully robot-assisted procedure for the radical treatment of pCCA patients at high-volume European tertiary center for HPB surgery. The present preliminary study will be useful for the purpose of designing a formal, prospective case–control study aimed at assessing the procedure safety and efficacy.

## Patients and methods

This study concerns 4 patients with a preoperative diagnosis of pCCA enrolled between March 2019 and March 2020. Inclusion and exclusion criteria for robotic surgery are shown in Table 1. During this same time frame, a total of 15 patients with suspected pCCA were selected for surgery at our center. All patients fulfilling the above-mentioned inclusion/exclusion criteria underwent the robotic approach.

**Table 1:** Selection criteria

Inclusion criteria	Exclusion criteria
Planned left hepatectomy	Diagnosis of cirrhosis
Absence of direct/indirect radiological signs of vascular invasion (portal/artery)	Evidence of major cardiovascular comorbidities, ASA score > III
Absence of previous major upper abdominal surgery	BMI > 30 kg/m^2^ (added after treating case #3)
Absence of previous hilar radiotherapy	Relevant anatomic variations (e.g. the left pedicle arising from the right anterior pedicle)
Informed consent to the robotic procedure	Need for a right hepatectomy

Table 2 shows the preoperative clinical characteristics of the patients undergoing fully robotic left hepatectomy including biliary carrefour and segment 1 en-bloc resection, followed by hepatico-jejunostomy. All patients presented preoperative biliary dilation, and all but one were treated with preoperative biliary drainage. We adopted the Bismuth classification for pCCA [15]. One patient received preoperative chemotherapy.

**Table 2:** Clinical characteristics

#	Age	Sex	Preoperative indication	Comorbidites	Bile duct dilation	Preoperative biliary tract management	Preoperative cholangitis	Preoperative chemotherapy	Preoperative bilirubin (mg/dL)
1	44	F	pCCA Bismuth 3b	None	Yes	Biliary stenting (ERCP)	No	Yes	4.21
2	61	M	pCCA Bismuth 3b	None	Yes	Biliary stenting (ERCP)	No	No	7.08
3	79	F	pCCA Bismuth 3b	Diabetes, hypertension	Yes	Biliary drainage (PTBD)	Yes	No	1.37
4	58	F	pCCA Bismuth 3b	None	Yes	Biliary drainage (PTBD)	Yes	No	6.02

*pCCA* perihilar cholangiocarcinoma, *ERCP* endoscopic retrograde cholangiopancreatography, *PTBD* percutaneous transhepatic biliary drainage

The preoperative diagnostic work-up included a triphasic computerized tomography (CT) scan of the chest, abdomen and pelvis in all cases, and magnetic resonance imaging with cholangio-pancreatography in 3 patients. There were strong clinical and radiological grounds for suspecting cancer in all patients. A positive preoperative biopsy was obtained in one patient. Brushing cytology during biliary drain placement was used for the other 3 patients, and a positive result was obtained for one of them. In short, a confirmatory biopsy was obtained preoperatively for 2 of the 4 patients.

### Surgical procedure (see attached video)

Robotic surgery was performed using the da Vinci Xi platform (Intuitive Surgical Inc., Sunnyvale, CA) with two active consoles, one used by the first surgeon, the other used occasionally and momentarily during the procedure by an assistant to control one robotic arm (e.g. during the biliary anastomosis, the assistant kept the suture taut between stitches). Trainees were only allowed to use the second console to watch the procedure (not to use the controls), to benefit from the 3D vision. One assistant constantly scrubbed in the operating field, while another also scrubbed to facilitate trocar positioning. Patients were placed supine with their legs apart, in a 30° reverse Trendelenburg position. Pneumoperitoneum was usually created with either a Verres needle or an open technique. All 4 robotic trocars were always used. The pneumoperitoneum was maintained with AirSeal^™^ at 15 mmHg. The 12 mm AirSeal^™^ trocars, and up to 3 other 5 mm trocars were used by the assistant for suction or exposure.

**Fig. 1:**
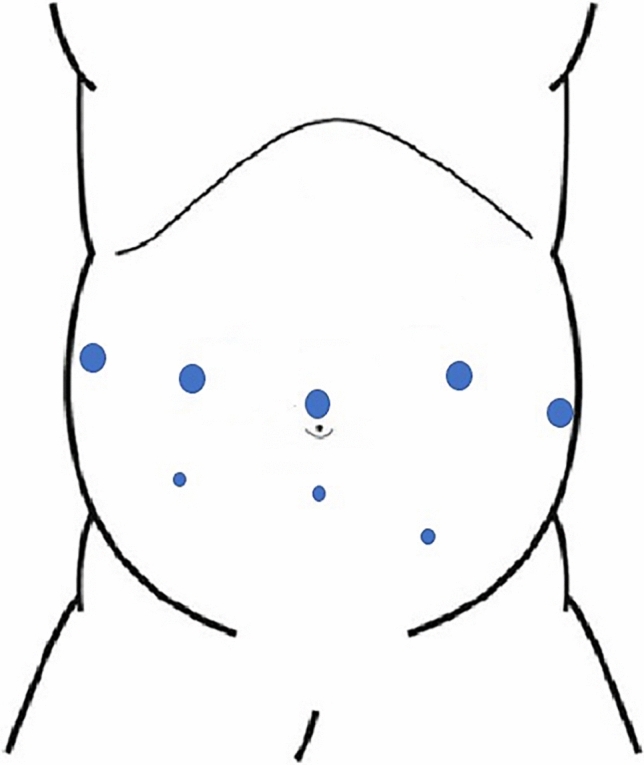
Trocar placement

The robotic trocars were usually placed horizontally along the imaginary umbilical line. The assistant placed laparoscopic trocars caudally between the robotic trocars (Fig. 1).

#### Hilar dissection, lymphadenectomy and liver resection

All the procedures began with a complete laparoscopic exploration of the abdomen.

After robotic platform docking, a complete lymphadenectomy was routinely performed, skeletonizing the hepatic artery as far as the celiac trunk.

In all cases, the distal common bile duct was divided with robotic scissors and oversewn. A stay suture was applied on the proximal choledochal stump to lift it up and improve the exposure of the hepatic hilum. The left and middle hepatic arteries (when present) were transected between Hem-o-loks^®^. The portal vein was usually ligated and robotically oversewn at the bifurcation. The Robotic Harmonic scalpel (Intuitive Surgical, Sunnyvale, CA) was used for parenchymal transection, and bipolar robotic forceps and Hem-o-loks^®^ for the major vascular structures. Preparations for the Pringle maneuver (extracorporeal clamping) were made in all cases, using an extra trocar positioned laterally in the left hypochondrium. The intrahepatic biliary ducts were usually dissected and sectioned proximally to the confluence after complete parenchymal transection for maximal exposure. Frozen sections of biliary stumps were routinely sent for pathology. Hepatic veins were stapled or sectioned between hem-o-loks. After complete hemostasis, the specimen was removed through a small midline incision, or a Pfannestiel incision in one case.

#### Biliary reconstruction

A Roux-en-Y loop was used for biliary reconstruction. For the sake of time in this preliminary phase, the loop was fashioned through the incision used to extract the specimen. Clearly, this technical step has the potential to be converted to mini-invasive approach once the overall time of the procedure will be reduced by expertise. The jejunum was divided with a stapler 50 cm from the Treitz angle. A latero-lateral jejuno-jejunostomy was created using a stapler. Pneumoperitoneum was restored and the loop was pulled towards the liver in a retro-colic fashion. The technique used for the hepatico-jejunostomy consisted of a single-layer 5–0 expanded tetrafluoroethylene (e-PTFE, Gore-Tex^©^) running suture. Lapra-ty^®^ clips (Ethicon) were used to secure both ends of the suture (see video). The jejunal loop was then secured to the liver serosa with interrupted stitches to avoid traction on the anastomoses.

## Results

Table 3 shows details of the surgical procedures, all four patients received a left hepatectomy with biliary resection and hepatico-jejunostomy. In Patient #2 the robotic procedure was converted to open surgery after extracting the specimen due to a short mesentery preventing the jejunal loop from being pulled up enough for a tension-free anastomosis. After the resection itself had been completed with the robotic procedure in this patient, the midline mini-incision was extended, and the biliary reconstruction involved a hepatico-gastrostomy instead of hepatico-jejunostomy as it proved impossible to pull up the jejunum even in the open setting.

**Table 3:** Details of the surgical procedures

#	Liver resection	Number of ducts (# of biliary anastomoses)	EBL (ml)	Blood transfusion	Pringle time (min)	Surgical time (min)	Conversion to open surgery	Reason for conversion
1	Left hepatectomy, biliary resection and hepatico-jejunostomy	2	700	No	0	770	No	
2	Left hepatectomy, biliary resection and hepatico-gastrostomy	3 (2)	800	No	30	950	Yes	Short mesentery
3	Left hepatectomy, biliary resection and hepatico-jejunostomy	1	600	No	27	790	No	
4	Left hepatectomy, biliary resection and hepatico-jejunostomy	5 (2)	700	No	20	890	No	

*EBL* estimated blood losses

**Fig. 2:**
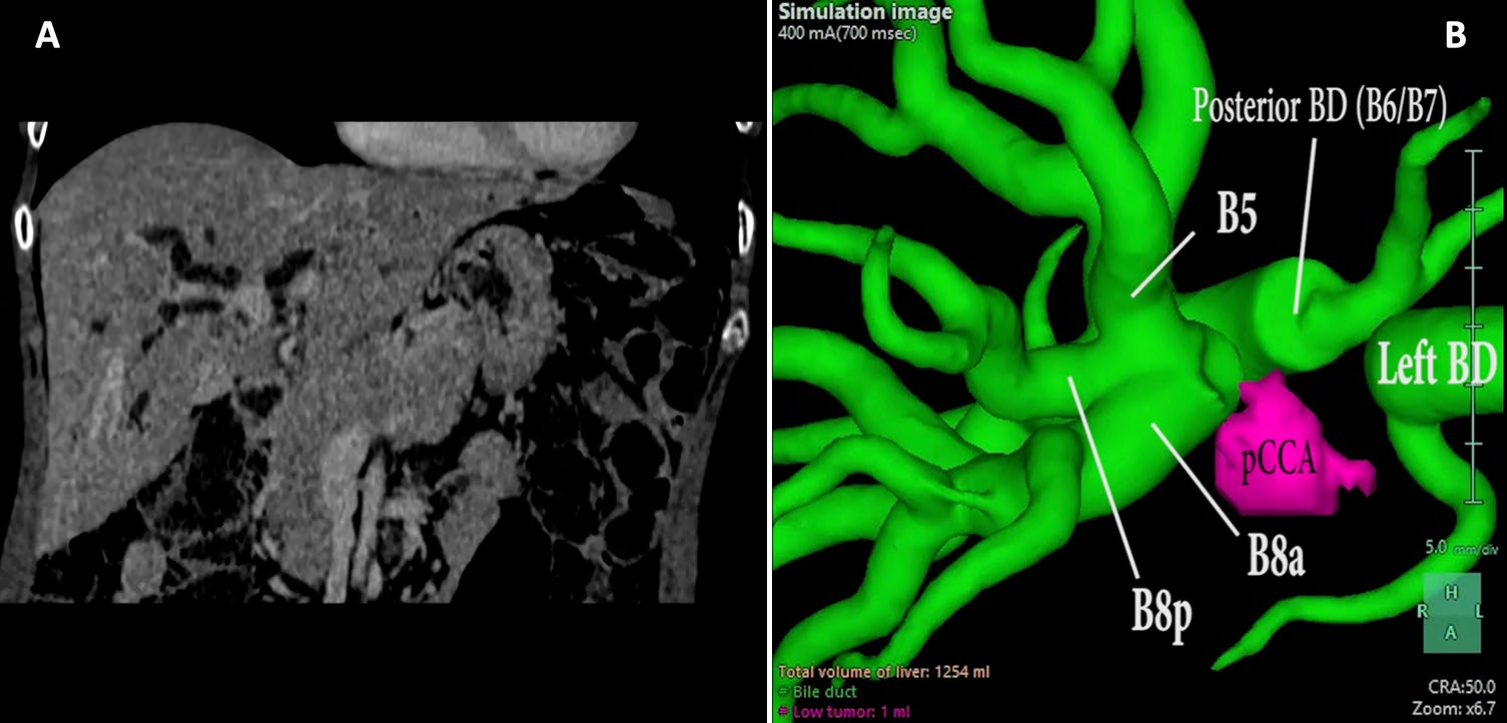
Preoperative CT scan of patient 4 (**a**); 3D reconstruction of the biliary tree of patient 4 (**b**)

**Fig. 3:**
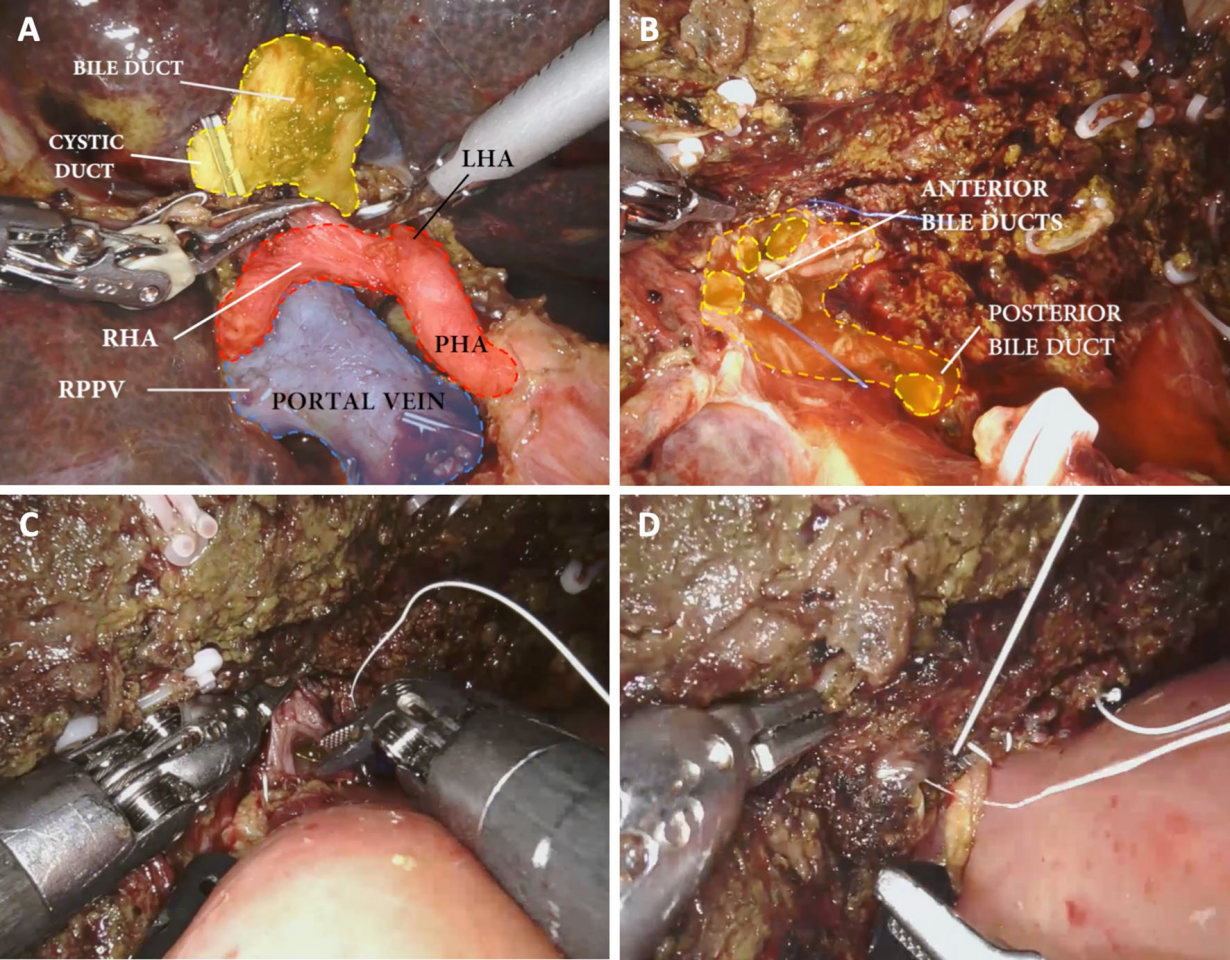
Intraoperative images (patient 4); exposure of the hilar plate (**a**) bile ducts before teatraduct hepatico-jejunostomy (3 anterior ducts, 1 posterior duct) (**b**); performing hepatico-jejunostomy, anterior layer (**c**), posterior layer (**d**)

The median time taken to complete the surgical procedures, from field preparation to skin closure, was long, a median 840 min (range 750–950). A maximum of 3 hilar clamping were used in 2 cases, with a maximum Pringle time of 30 min. No Pringle maneuvers were used in the other 2 cases. As mentioned, the biliary reconstruction was managed robotically in 3 of the 4 cases. It involved one duct in one case, two ducts in another, and in the third (Fig. 2) we found 5 biliary stumps after the transection, so the reconstruction was done with two different running sutures, including two stumps in one and three in the other (see video and Fig. 3). None of the patients required blood transfusions intraoperatively or during their hospital stay.

The postoperative course was favorable in all cases. We recorded no major complications (Dindo Clavien > 3) [16]. There was one biliary leak, which was grade A according to the International Study Group of Liver Surgery [17]. Other complications included: a postoperative ileus (managed and resolved conservatively); and an asymptomatic segmental ischemia (segment 5), documented on a postoperative scan. The median postoperative hospital stay was 9 days. Final pathology confirmed the preoperative clinical suspicion in all cases. Negative biliary margins were achieved in 3 of the 4 patients.

Up to 14 lymph nodes were retrieved for pathology from three patients, while none were found at final pathology for Patient #1. This patient had been administered preoperative chemotherapy with a massive response. The hilar structures were extremely difficult to dissect, probably due to scarring caused by this extensive response to chemotherapy. The hilum was approached as usual, lifting the common bile duct and removing all the tissue around the vessels to completely expose the portal vein and hepatic arteries. The hilar soft tissue was sent en bloc with the liver specimen for final pathology. Multiple tissue specimens obtained along the proper hepatic artery were sent for frozen analysis, and all came back as non-neoplastic fibrous tissue. The recurrence pattern, in this case, was multifocal in multiple sites: liver, bone and cervical lymph nodes two months after surgery.

At short-term follow-up (see the last two columns of Table 4), we have had 1 case of recurrence; the patient is alive with disease. The other 3 patients are alive and well, with no evidence of disease.

**Table 4:** Postoperative course, pathologist’s findings, and short-term follow-up

#	Postoperative hospital stay (days)	Complications (Dindo-Clavien) [16]	Type of complication	Biliary margins	Final staging	Follow-up (months)	Status at latest follow-up
1	11	2	Ileus	Negative	T3Nx	12	AWD
2	10	2	Biliary leak (grade A)	Positive, proximal	T2aN1	8	NED
3	8	0	–	Negative	T4N1	7	NED
4	7	1	Asymptomatic segment 5 ischemia	Negative	T4N0	6	NED

*AWD* Alive with disease, *NED* No evidence of disease

## Discussion

Recommended surgery for pCCA includes complete resection of the extrahepatic bile duct, extended hemi-hepatectomy, liver-hilum lymphadenectomy and hepatico-jejunostomy [1]. Today, open surgery is still recognized as the standard approach to radical treatment in this setting. On the other hand, advantages of minimally-invasive liver surgery have been widely reported, in the oncologic setting as well. [6, 18–20].

Though it was introduced later on, robot-assisted liver surgery is now practiced at many institutions around the world [21], but there is still a substantial shortage of evidence clearly addressing the issue of which specific liver procedures are worth managing with a robotic approach and in a number of particular fields its adoption is still under debate [22, 23].

In the particular setting of pCCA, biliary reconstruction can be demanding in laparoscopy, especially in the case of small ducts or multiple biliary stumps. Previous studies on minimally-invasive procedures have demonstrated the feasibility of managing pCCA laparoscopically with the limitation, so far, of ductal anastomoses [24]. The robotic approach has the well-known advantages of a 3D view and more flexible movements. Technically enhanced ‘wristed’ robotic instruments with additional degrees of freedom and finer motion scaling control enable surgeons to undertake challenging maneuvers, including small anastomoses, with optimal surgical dexterity [25, 26]. These features provide the absolutely novel chance to perform also minimally-invasive hepatico-jejunostomies; even in presence of multiple ducts. The attached video showing a tri-ductal hepatico-jejunostomy clearly points to these advantages. The robotic approach has, therefore, the intrinsic potential to manage the whole procedure mini-invasively. Indeed, in this exploratory phase, due to the prolonged time and the complexity of the crucial parts of the procedure we decided, for the sake of time, to proceed with a minilaparotomy to prepare the intestinal loop and to extract the specimen. Clearly, at a later development stage, it will be possible to complete this step robotically and the specimen extraction will be performed through a Pfannestiel incision.

Even though the first robotic liver resection for pCCA was reported in 2010 by Giulianotti et al. [10], since then, only a few case reports have appeared in the literature and exclusively from the east, at the best of our knowledge (Table 5). The first series of robotic approach for pCCA was published in 2012 [12]. Liu et al. reported on 39 cases. The authors report the total number of procedures performed for pCCA, but not how many of them involved fully robotic hepatectomies with biliary reconstructions. They somewhat vaguely describe the procedure on the liver as “excision of tumor”, with biliary reconstruction in 36 cases, and they report 3 cases of left hemi-hepatectomy (1 converted to open surgery). Other data are reported together with details of procedures performed for other biliary malignancies, preventing us from drawing any meaningful conclusion.

**Table 5:** Postoperative course, pathological findings and short-term follow-up in published case reports and case series

Author	Year	# of cases	Bismuth type (I:II:III:IV)	MH	LN	Operating time (min)	Blood loss (mL)	Conversion (%)	R0/R1	POD (days)	Complications (%)	30-day mortality (%)
Giulianotti et al. [10]	2010	1	0:0:1:0	1	1	540	800	0	1/0	11	0	0
Liu et al. [12]	2012	39	1:8:14:16 *	3	NA	190–650^§^	NA	2.6	NA	NA	14.1	2.6
Zhu et al. [27]	2014	1	0:0:1:0	1	NA	NA	700	0	1/0	14	0	0
Xu et al. [11]	2016	10	0:1:5:4	10	10	703 (600–800)^§§^	1360 (400–3000)	0	5/3**	18 (9–58)^#^	90	10
Chong et al. [28]	2019	1	NA	1	1	510	360	0	1/0	16	0	0
Li et al. [14]	2020	48	20:6:22:0	NA	48	276 (170–500)^#^	150 (20–1500)	NA	35/13	9 (4–52)	58.3	0
Machado et al. [29]	2020	1	0:0:1:0	1		480	740	0	R0	NA	Abdominal collection	0
Marino et al. [30]	2020	1	0:0:1:0	1	9	280	280	0	NA	6	None	0
Cillo et al. (present series)	2020	4	0:0:4:0	4	4	840 (770–890)	700 (600–800)	25	3/1	9 (7–11)	75	0

*MH* major hepatectomy, *LN* lymphadenectomy, *POD* postoperative hospital stay, *NA* not applicable

**Data are missing for 2 cases

^#^The author reports the time the surgeon spent at the console

In 2012 Li et al. [13] reported on the most numerous series published to date, including 48 robotic surgical procedures for pCCA. Here again, however, the report lacks many important details about the type and extent of the tumor, the resection and biliary reconstruction procedures, the conversion rate, the total operating times and more, making insufficient the informative content.

On the contrary, the series subsequently reported by Xu et. al. [11] on 10 robotic procedures for pCCA, mostly Bismuth types 3 and 4, provide precise information about operating times, blood loss, and postoperative outcome. As in our case series, Xu et al. describe long operating times (median 700 min compared to 840 in our series). Intraoperative blood loss was apparently higher than in our cohort (1300 ml versus 700 ml), but no conversions were reported. As for the morbidity profile, we had no major complications, and only 1 biliary leak, whereas Xu et al. reported that 3 out of 10 patients had Dindo-Clavien > 3 complications. Our patients had a median postoperative hospital stay of 9 days, which is shorter than the median 16 days reported by Xu et al. From an oncological standpoint, surgical margins were negative in 3 of our 4 cases, while Xu et al. reported positive proximal biliary margins in 3 of their 10 patients (and they do not provide the data on 2 cases).

Judging from a recent systematic review [14], no other case series have been published, but only case reports.

At this stage, the previously reported results of robotic treatment of pCCA and our herein presented experience, are too preliminary and inhomogeneous to draw conclusions as far as safety, efficacy and oncologic radicality are concerned. For similar reasons, single events as conversion and R1 resection in our series have to be seen through the lens of an initial part of a learning curve in the context of an extremely complex procedure. So is for the long operative times. In this perspective, the absence of perioperative mortality and severe complications are promising but clearly not definitive.

In Patient #2 the robotic procedure was converted to open surgery due to a short mesentery preventing the jejunal loop from being pulled up enough for a tension-free anastomosis. It was a frank mistake of pre and intraoperative evaluation. Indeed, the patient was overweight, presenting visceral obesity. After the case, we excluded antropometrically similar patients from this preliminary robotic procedure. Luckily enough, no hepatico-gastrostomy associated complications were recorded during the 12 months of patient follow-up.

Our pilot study was conducted in the context of a center with lengthy experience of HPB surgery. Without a relevant HPB technical background, it would be totally unadvisable to attempt minimally invasive approaches in pCCA.

Although our preliminary results are encouraging, in terms of demonstrating the feasibility of the procedure, this case series has the obvious limitations typical of preliminary experiences. Again, the small number of cases, and the unability of conducting a comparative analysis hamper the possibility to conclude that the robotic approach for pCCA is safe and effective at this stage, in particular as far as the oncologic results are concerned. Our initial feasibility experience, nonetheless, besides giving technical insights through the availability of an included video clip, paves the way to numerically more relevant, prospective comparative studies, focusing on the safety and long-term oncological results of robot-assisted surgery for pCCA.

## Supplementary Information

Below is the link to the electronic supplementary material.

Supplementary file1 (MP4 261636 KB)

## Data Availability

The datasets generated during and/or analyzed during the current study are available from the corresponding author on reasonable request.
